# The role of right ventricular volumes and inferior vena cava diameters in the evaluation of volume status before colonoscopy

**DOI:** 10.3906/sag-1903-98

**Published:** 2019-12-16

**Authors:** Sule ARICAN, Ramazan DERTLİ, Çağdaş DAĞLI, Gülçin HACIBEYOĞLU, Mustafa KOYUNCU, Ahmet TOPAL, Sema TUNCER UZUN, Mehmet ASIL

**Affiliations:** 1 Department of Anesthesiology, Meram Faculty of Medicine, Necmettin Erbakan University, Konya Turkey; 2 Department of Internal Medicine, Division of Gastroenterology, Meram Faculty of Medicine, Necmettin Erbakan University, Konya Turkey

**Keywords:** Colonoscopy, hypotension, ultrasonography

## Abstract

**Background/aim:**

Ultrasonographic measurements of inferior vena cava (IVC) diameters and right ventricle (RV) volumes are important tools for the evaluation of intravascular volume. The current study investigates the association of IVC diameters and RV volumes before colonoscopy in prediction of postanesthesia hypotension.

**Materials and methods:**

Seventy patients scheduled for colonoscopy were included in the study. Preoperatively, expirium (dIVC max) and inspirium (dIVC min) IVC diameters were measured using M-mode ultrasonography and the collapsibility index (IVC-CI) was calculated. Ventricular volumes and areas were also measured using transthoracic echocardiography. Postanesthesia hypotension was defined as mean arterial blood pressure of <60 mmHg or a decrease of >30% in the mean arterial pressure after sedation.

**Results:**

Minimum and maximum IVC diameters were significantly lower (P = 0.005 and P < 0.001, respectively) and IVC-CI was significantly higher (P < 0.001) in patients who developed hypotension. Similarly, right ventricular end-diastolic area (RV-EDA), right ventricular end-systolic area (RV-ESA), right ventricular end-diastolic volume (RV-EDV), right ventricular end-systolic volume (RV-ESV), and left ventricular end-systolic volume (LV-ESV) values were significantly lower in patients with hypotension (P < 0.05). Logistic regression analysis showed that dIVC min and RV-ESA were independent predictors of hypotension.

**Conclusion:**

IVC diameters and RV-ESA, RV-EDA, RV-ESV, and RV-EDV are good indicators of preoperative volume status and can be used to predict the patients at risk of developing hypotension.

## 1. Introduction

Anesthesia may cause hemodynamic instability and hypotension in the perioperative period. Perioperative blood pressure instability is clinically important because it is associated with cardiac, renal, and neurologic adverse events [1,2]. Volume status has a major impact on the maintenance of perioperative hemodynamic homeostasis; therefore, accurate evaluation prior to operation is important. Several patient- and procedure-related factors, such as physical status of the patient, comorbidities, and preoperative interventions such as bowel preparation and long fasting duration, may affect preoperative volume status [3]. Various methods have been described for accurate preoperative estimation of volume status, and methods used to evaluate volume status have evolved from static pressure and volume parameters to dynamic indices [4].

Ultrasonographic measurements of inferior vena cava (IVC) diameters and right ventricular (RV) volumes are important tools in the evaluation of preoperative intravascular volume status and response to fluid therapy [5]. Hand-carried cardiac ultrasound (HCU) can be used for these measurements, and there are acceptable accuracy rates in cardiac evaluation of these parameters by noncardiologists [6,7]. Transthoracic echocardiography (TTE) enables rapid evaluation of anesthetized and awake patients and eliminates the need for invasive monitorization of the circulation status [2]. Preoperative respiratory variation of IVC has been shown to predict hypotension after induction under general anesthesia with high sensitivity and specificity [8]. In this context, the objective of the current study was to evaluate the predictive power and correlation of USG measurements of vena cava inferior diameters and right ventricular volumes for prediction of postanesthesia hypotension in patients undergoing colonoscopy with sedoanalgesia.

## 2. Materials and methods

### 2.1. Patients

This study was conducted in the gastroenterology outpatient clinic of the Necmettin Erbakan University Meram Faculty of Medicine Hospital as a single-center observational study between January 2018 and August 2018 after receiving the necessary approval from the local ethics committee, in accordance with the Declaration of Helsinki. The participants were informed about the study in detail both verbally and in writing, and all patients provided informed consent. A total of 70 patients aged over 18 years with an American Society of Anesthesiologists (ASA) physical status classification of I–III who were scheduled for colonoscopy under sedation were included in the study. All patients underwent bowel preparation before the operation. This preparation entailed a clear diet in all patients 2 days before the operation, a laxative solution containing 20 mL of sennoside A-B and calcium salt (X-M Solution laxative, 250 mL, Yenişehir Laboratuar Ticaret ve Sanayi Şti, Turkey) 24 h before the operation, and a watered enema given in 8-h intervals. Patients were told that they could drink particle-free clear fluid until 3 h before the procedure. Patients with increased intraabdominal pressure, heart failure, valvular disease, portal hypertension, a difficult airway, or chronic obstructive pulmonary disease; those using diuretics; pregnant patients; and those with peripheral vascular disease, autonomic nervous system disease, mental disorders, or a history of pulmonary hypertension were excluded from the study. Demographic data (age, sex, height, weight, and body mass index), ASA classification, and preoperative fasting times were recorded for all patients. After routine monitorization, baseline values of blood pressure, heart rate, and peripheral oxygen saturation were recorded. After the procedure, the Modified Aldrete Score (MAS) was used to measure the recovery. Patients were discharged to their homes when the MAS was 10, and the time it took for each patient to reach a MAS score of 10 after the procedure was recorded. 

### 2.2. Ultrasonography imaging

Ultrasonographic IVC measurements were performed while patients were in a supine position before colonoscopy with a Mindray ultrasound device. Measurements were obtained in the abdominal mode and a sector probe was used. IVC ultrasonography was performed for each patient according to the methodology described by the American Echocardiography Society, with a subcostal approach using an intermediate median long-axis image. A two-dimensional IVC image was acquired beginning from the right atrium, and respiratory changes in IVC diameters were gathered 2–3 cm distal to the right atrium. Expirium (dIVC max) and inspirium (dIVC min) diameters were measured at least 3 times in M-mode and the collapsibility index (IVC-CI) was calculated according to the formula IVC-CI = (dIVC max – dIVC min) / IVC max × 100. Data of patients were excluded if there was a difference higher than 0.2 cm in dIVC max measurements between any 2 images. The USG mode was then changed to heart mode and patients were moved to a left decubitus position. Apical imaging of the RV and left ventricle (LV) was obtained using harmonic imaging with a transthoracic 3-MHz phase sector transducer (Mindray M7 ultrasound device). The end-systolic area (ESA) and end-diastolic area (EDA) were measured for both right and left ventricles. The volumes of the cardiac chambers were assessed with the Simpson method. The RV end-diastolic volume (EDV), RV end-systolic volume (ESV), and ejection fraction (EF) measurements were obtained using software loaded to the ultrasonography device. The EDV was measured when the tricuspid valve was closed, and ESV was measured via the smallest RV chamber image. Both measurements were made from the apical four-chamber view by tracing the endocardial margin of the RV. The two-dimensional echocardiography subtraction method was obtained from the apical four-chamber view by tracing the volume of the LV with the inclusion of the interventricular septum and subtracting it from the total volume of the LV and RV. 

### 2.3. Anesthesia management

After these measurements were taken, standard deep sedation (Ramsey sedation score: 5–6) was performed by an anesthetist who was not involved in the study. Each patient was administered 0.01–0.03 mg/kg midazolam, 0.1–1 µg/kg fentanyl, and 1–2 mg/kg propofol. Additional doses were administered during the operation as needed. The agents and doses used in sedation were recorded. Routine monitorization continued in the operating room. Blood pressure measurements were performed using a noninvasive oscillometric method. Patients’ heart rate, blood pressure, and peripheral oxygen saturation (SpO2) measurements were assessed every 2 min from sedation until the end of the colonoscopy procedure. Postanesthesia hypotension was defined as mean arterial pressure (MAP) of <60 mmHg or a decrease of >30% in MAP after sedation.

### 2.4. Statistical analysis

Results of the study were analyzed with SPSS 19.0 for Windows (IBM Corp., Armonk, NY, USA). Continuous variables were expressed as mean ± standard deviation and categorical variables as number and percentage (n, %). Normal distribution of the data was analyzed with the Kolmogorov–Smirnov test, histograms, and ±SD. Nonparametric data of the groups were compared with the Mann–Whitney U test and parametric data with the independent sample t-test. Categorical data were analyzed with the chi-square test. ROC curve analyses were performed in order to test the ability of preoperative dIVC max, dIVC min, IVC-CI, RV-ESA, RV-EDA, RV-ESV, and RV-EDV in prediction of clinically significant hypotension in the perioperative period. Sensitivity, specificity, and positive and negative predictive values were calculated for optimal cut-off values. Correlation between hypotension and ultrasonographic data was evaluated with Spearman’s correlation analysis. P < 0.05 was considered statistically significant.

## 3. Results

### 3.1. Patients’ demographics and hemodynamic data

A total of 70 patients scheduled for colonoscopy under sedation were included in the study. The mean age of the patients was 49.9 ± 15.03 (19–79) years, and the male/female ratio was 32/38 (45.7% vs. 54.3%). Eighteen (25.7%) patients developed hypotension after sedation. Demographic and baseline hemodynamic data of the whole study group and demographic and hemodynamic data of the patients who developed hypotension after sedation are shown in Table 1. Demographic characteristics and baseline hemodynamic data were found to be similar in patients with and without hypotension (P > 0.05). On the other hand, a significant difference was found between these 2 groups in terms of systolic arterial blood pressure (SAP), diastolic arterial blood pressure (DAP), and MAP assessed after sedation, as would be expected (P < 0.05). Fasting durations were also statistically significantly longer in the patients who developed hypotension (P = 0.04). Doses of hypnoid and opioid drugs used for sedation were found to be similar between the patients with and without hypotension (P > 0.05).

**Table 1:** Demographic and baseline hemodynamic data of the patients.

	Hypotension			
	No (n: 52)	Yes (n: 18)	Total (n: 70)	P-value
Age (years)	51.26 ± 14.42	46.11 ± 16.49	49.90 ± 15.03	0.212
Sex (male/female)	24/28	8/10	32/38	0.561
Body length (cm)	165.34 ± 8.02	165.44 ± 7.78	165.7 ± 7.91	0.964
Body weight (kg)	73.44 ± 14.11	68.16 ± 9.6	72.08 ± 13.24	0.147
BMI (kg/m^2^)	26.92 ± 5.28	24.99 ± 3.80	26.43 ± 4.99	0.159
ASA I/II/III	8/35/9	3/11/4	11/46/13	0.875
Fasting time (h)	8.03 ± 2.95	10.72 ± 4.15	8.72 ± 3.48	0.04*
Baseline values				
SAP, mmHg	139.73 ± 20.12	135.38 ± 19.39	138.61 ± 19.89	0.429
DAP, mmHg	76.00 ± 12.81	79.44 ± 11.31	76.88 ± 12.46	0.316
MAP, mmHg	97.23 ± 13.46	98.11 ± 12.58	97.45 ± 13.15	0.809
HR, beats/min	86.72 ± 15.00	92.55 ± 14.29	88.25 ± 14.94	0.158
SpO_2_, %	95.40 ± 4.91	95.88 ± 16.52	95.52 ± 4.43	0.693
Postinduction values				
SAP, mmHg	115.15 ± 18.87	98.83 ± 16.52	110.95 ± 19.55	0.002*
DAP, mmHg	65.94 ± 12.48	53.22 ± 7.19	62.67 ± 12.62	P < 0.001*
MAP, mmHg	83.16 ± 13.63	66.88 ± 8.27	78.98 ± 14.34	P < 0.001*
HR, beats/min	77.94 ± 13.58	77.50 ± 7.4	77.82 ± 12.25	0.896
% SpO_2_	97.13 ± 2.62	97.72 ± 1.87	97.28 ± 2.45	0.386
MAS 10 (min)	10.44 ± 2.23	11.33 ± 2.72	10.67 ± 2.38	0.173
Hypnotics and opioids				
Midazolam	1.25 ± 0.42	1.41 ± 0.46	1.30 ± 0.43	0.191
Fentanyl	61.4 ± 19.56	69.4 ± 23.5	63.5 ± 20.78	0.161
Propofol	70.86 ± 37.7	67.2 ± 31.0	69.92 ± 35.94	0.714

BMI = Body mass index; ASA = American Society of Anesthesiologists physical status; SAP =systolic blood pressure; DAP = diastolic blood pressure; MAP = mean blood pressure; HR = heart rate; SpO_2_ = peripheral oxygen pressure; MAS (min) = time (in minutes) for patients to reach a Modified Aldrete Score of 10 after the procedure. *P < 0.05.

### 3.2. Ultrasonographic measurement data

In USG evaluation, IVC diameters were significantly lower and IVC-CI was significantly higher in the patients who developed hypotension (P < 0.05). Similarly, in the evaluation of cardiac chambers, RV-EDA, RV-ESA, RV-EDV, RV-ESV, and LV-ESV values were found to be significantly lower in the patients who developed hypotension (P < 0.05). Preprocedural ultrasound measurements of cardiac chambers and IVC diameters in patients with and without hypotension are summarized in Table 2. 

**Table 2:** Preprocedural ultrasound measurements of cardiac chambers and IVC diameters.

	Hypotension			
	Yes (n: 18)	No (n: 52)	Total (n: 70)	P-value
dIVC max, cm	1.30 ± 0.31	1.65 ± 0.47	1.56 ± 0.46	0.005*
dIVC min, cm	0.61 ± 0.23	1.17 ± 0.43	1.02 ± 0.46	P < 0.001*
IVC-CI, %	0.5 ± 0.14	0.3 ± 0.12	0.36 ± 0.16	P < 0.001*
RV-ESA, cm^2^	6.1 ± 1.86	7.7 ± 1.91	7.35 ± 2.02	0.002*
RV-EDA, cm^2^	10.7 ± 2.48	12.7 ± 2.60	12.24 ± 2.70	0.006*
RV-ESV, ml	7.4 ± 3.4	9.4 ± 3.2	8.98 ± 3.36	0.028*
RV-EDV, ml	17.3 ± 5.8	20.7 ± 6.3	19.8 ± 6.36	0.044*
RV-FAC	0.41 ± 0.17	0.38 ± 0.10	0.39 ± 0.12	0.396
LV-ESA, cm^2^	15.2 ± 2.81	16.7 ± 3.7	16.3 ± 3.56	0.131
LV-EDA, cm^2^	26.8 ± 4.94	29.0 ± 5.1	28.4 ± 5.13	0.121
LV-ESV, ml	32.5 ± 6.0	39.4 ± 13.0	37.68 ± 12.05	0.034*
LV-EDV, ml	83.1 ± 21.6	94.2 ± 26.0	91.4 ± 25.3	0.108
EF, %	60.4 ± 5.5	58.4 ± 6.6	58.0 ± 0.64	0.267
Stroke volume	4.6 ± 1.75	4.7 ± 1.56	4.74 ± 1.60	0.816

dIVC max = Maximum diameter of IVC; dIVC min = minimum diameter of IVC; CI = collapsibility index; RV = right ventricle; ESA = end-systolic area; EDA = end-diastolic area; ESV = end-systolic volume; EDV = end-diastolic volume; FAC = fractional area change; LV = left ventricle; EF = ejection fraction. *P < 0.05.

### 3.3. Prediction of hypotension

ROC analyses were carried out for each of the measured parameters to test the ability to predict hypotension developing after sedation. Among tested parameters, the best results were obtained with IVC-CI and dIVC, both of which showed good diagnostic accuracy. For IVC-CI, the calculated AUC was 0.854 (P < 0.001, 95% CI: 0.743–0.966), and the specified optimal cut-off value of 45% yielded 83.3% sensitivity and 82.7% specificity. For this cut-off value, positive and negative predictive values were 62.5% and 93.5%, respectively. For dIVC, the AUC was 0.866 (P < 0.001, 95% CI: 0.783–0.950). The optimal cut-off value of dIVC min was found to be 1 cm, with 94.4% sensitivity and 71.2% specificity. Calculated positive and negative predictive values were 53.1% and 97.3%, respectively. ROC analyses are summarized in Table 3.

**Table 3:** Prediction of hypotension, ROC analyses.

	AUC	Cut-off	95% CI	Sensitivity	Specificity	+ Predictive value	-Predictive value	P-value
dIVC max	0.736	1.515	0.612–0.859	77.8	61.5	41.17	88.9	0.03
dIVC min	0.866	1.005	0.783–0.950	94.4	71.7	53.1	97.3	P < 0.001
IVC-CI	0.854	0.45	0.743–0.966	83.3	82.7	62.5	93.5	P < 0.001
RV-ESA	0.738	5.95	0.595–0.881	61.1	82.7	55	86	0.03
RV-EDA	0.669	11.65	0.560–0.836	66.7	67.3	41.37	85.36	0.013
RV-ESV	0.663	7.26	0.513–0.814	61.1	67.3	39.28	83.33	0.040
RV-EDV	0.635	18.95	0.488–0.781	55.6	61.5	33.3	80	0.09

DIVC max = Maximum diameter of IVC; dIVC min = minimum diameter of IVC; CI = collapsibility index; RV= right ventricle; ESA = end-systolic area; EDA = end-diastolic area; ESV = end-systolic volume; EDV = end-diastolic volume; AUC; area under curve, 95% CI = confidence interval.

### 3.4. Correlation analysis

There was a strong positive correlation between hypotension and IVC-CI (r = 0.590, P < 0.001), while strong negative correlations were found between hypotension and dIVC max (r = –0.330, P = 0.005), dIVC min (r = –0.530, P < 0.001), RV-ESA (r = –0.363, P = 0.002), and RV-EDA (r = –0.328, P = 0.006) (Table 4).

**Table 4:** Correlation analyses of ultrasound parameters with hypotension.

Variable	R	P-value
dIVC max and hypotension	–0.330	0.005**
dIVC min and hypotension	–0.530	P < 0.001**
IVC-CI and hypotension	0.590	P < 0.001**
RV-ESA and hypotension	–0.363	0.002**
RV-EDA and hypotension	–0.328	0.006**
RV-EDV and hypotension	–0.242	0.044*
RV-ESV and hypotension	–0.263	0.028*
LV-ESV and hypotension	–0.254	0,034*

DIVC max = Maximum diameter of IVC; dIVC min= minimum diameter of IVC; CI = collapsibility index; RV= right ventricle; ESA = end-systolic area; EDA = end-diastolic area; ESV = end-systolic volume; EDV = end-diastolic volume; FAC = fractional area change; LV = left ventricle; EF = ejection fraction. *Correlation is significant at the 0.05 level, **correlation is significant at the 0.01 level.

### 3.5. Logistic regression analysis

Logistic regression analysis showed that dIVC min (OR: 0.015, P < 0.001, 95% CI: 0.02–0.131) and RV-ESA (OR: 0.585, P = 0.04, 95% CI: 0.406–0.844) were independent predictors of hypotension.

## 4. Discussion

In the present study, we found that ultrasonographic evaluation of IVC, RV-ESA, RV-EDA, RV-ESV, and RV-EDV before colonoscopy predicted and were correlated with hypotension developing after sedation. In clinical practice, routine monitorization of physiological parameters such as heart rate, arterial blood pressure, central venous pressure, peripheral oxygen saturation, and urine output is often used for assessment of intravascular volume and guide fluid therapy. However, the sensitivity and specificity of these parameters in determining subclinical hypovolemic or hypervolemic conditions are low. Therefore, the use of the static parameters described above in order to manage perioperative fluid therapy may cause hypovolemia or hypervolemia [9,10]. Similarly, in our study we found that there was no significant difference in baseline arterial pressure values between the patients who developed hypotension after sedation and those who stayed hemodynamically stable with no significant change in arterial blood pressure. It has been shown in previous studies that there is no correlation between static hemodynamic data such as central vein pressure and pulmonary arterial occlusion pressure and IVC measurements [9,11]. Therefore, we used measurements of IVC together with cardiac chambers in our study, and good correlation was observed between these 2 dynamic monitorization methods. There are some risk factors associated with the development of postanesthesia hypotension in patients undergoing colonoscopy. First of all, preoperative fasting and bowel preparation may cause hypovolemia [12]. Our findings are also consistent with this because the mean fasting time was significantly longer in patients who developed hypotension after sedation. 

IVC diameter is not affected by the compensatory vasoconstrictor response given by the body to volume loss, and it is a reliable indicator of blood loss even in the small quantity of 450 mL [13,14]. Previous studies have shown that ultrasonographic measurement of IVC diameter may be a rapid and noninvasive method for clinicians in the evaluation and management of critical patients [15,16]. Recent clinical studies have also demonstrated that the most reliable measurement of IVC diameter can be made at 2 cm caudal from the hepatic vein and IVC junction [17,18]. Therefore, in our study IVC diameters were assessed using this technique, and measurement errors were minimized. We observed that IVC diameters were effective in the prediction of hypotension, which may develop in patients under sedation, and optimal cut-off values were 45% for IVC-CI, 1.51 cm for IVC max, and 1.0 cm for IVC min. Our findings also showed that IVC min was an independent predictor of hypotension in regression analyses. Similar results were also reported in the literature [19, 20]. In a recent study, Salama et al. reported that IVC-CI was significantly higher in patients who developed postspinal anesthesia hypotension than in patients who did not [21]. Also, in the study conducted by Saranteas et al., it was found that preoperative dIVC max/IVC-CI predicted spinal-induced hypotension better than echocardiographic measurements [22]. 

Noninvasive transthoracic echocardiography (TTE) examination is possible using portable ultrasound systems [23–27]. This method has been used for the evaluation of critical patients since it enables rapid assessment of volume and contractility of both ventricles. TTE is helpful in screening serious pathologies, and it is generally considered sufficient in evaluation of cardiac function and volume load. Although it does not allow for a comprehensive cardiac examination, this screening may directly affect patient management in the perioperative period [27,28]. Our results are also in parallel with the literature in this aspect. The results of the current study showed that preoperative TTE examination and right ventricular measurements were important tools in the prediction of hypotension developing in the perioperative period. TTE examination can be considered as a simple and effective tool to evaluate the volume status of patients preoperatively, and it can be used in the perioperative management of patients. In the current study, diagnostic accuracy when using RV end-systolic and end-diastolic area and volumes was found to be lower compared to the use of IVC measurements, but there were still strong negative correlations between RV-ESA, RV-EDA, and hypotension. RV-ESA was also found to be an independent predictor of hypotension in the logistic regression analysis.

The use of perioperative/intraoperative echocardiography by an anesthesiologist may change the management of these patients and improve possible negative outcomes [29]. This method is an adjuvant technique for anesthesiologists in directing perioperative clinical management [30]. Kratz et al. showed that focused TTE performed by an anesthesiologist is an effective tool that provides important data in the hemodynamic management of unstable patients, and they encouraged the use of TTE in perioperative medicine to provide better and more sustainable care in particularly complex cases [31].

In our study, a considerable proportion of the patients (25.7%) developed hypotension under sedation, and noninvasive evaluation of IVC diameters and right cardiac chambers in these patients before sedation was found to be helpful in the prediction of possible hypotension. In this context, we believe that preoperative evaluation of IVC diameters and right cardiac chambers would guide hemodynamic management of the patients in the perioperative period, and therefore necessary measures can be taken early for patients with increased risk.

The current study has several limitations. First of all, all echocardiographic measures were made by an operator with experience at a basic level. However, we believe that this is not a critical issue because acceptable accuracy rates were reported in the literature in cardiac evaluation by noncardiologists using cardiac ultrasound [6,7]. It is also important to note that perioperative echocardiography performed by an anesthesiologist is not an alternative to detailed echocardiographic examination performed by experienced cardiologists; rather, it is an additional tool for the anesthesiologist in guiding perioperative management [31]. Second, the measurements were performed only before the operation and could not be made after sedation because of patient positioning and limited setting. Third, a single end-systolic and end-diastolic measurement of RV and LV areas and volumes with TTE may lead to faulty insight for recognition and evaluation of volume status due to dynamic variability. Therefore, serial measurements would be better in order to assess volume status more sensitively.

In conclusion, preoperative dynamic measurements of IVC diameters and right ventricular area and volumes are important tools for the evaluation of the preoperative volume status of patients undergoing colonoscopy with IV sedoanalgesia. These measurements can easily be obtained in outpatient clinical settings and can be used in prediction of postanesthesia hypotension. Preoperative measurement of CI and dIVC min predict the incidence of hypotension after induction with high sensitivity and specificity. Sensitivity and specificity are lower for RV measurements compared to IVC measurements, and RV measurements can be used in patients where IVC measurements are impossible or inconclusive. dIVC max, dIVC min, RV-ESA, and RV-EDA are correlated with hypotension, and dIVC min and RV-ESA are independent predictors of hypotension. 

Further studies are needed to determine fluid therapy strategies based on the measurements of IVC/RV areas and volumes in order to prevent postanesthesia hypotension.
